# MiXie, an Online Tool for Better Health Assessment of Workers Exposed to Multiple Chemicals

**DOI:** 10.3390/ijerph19020951

**Published:** 2022-01-15

**Authors:** Bénédicte La Rocca, Philippe Sarazin

**Affiliations:** 1 Toxicology and Biometrology Department, French National Research and Safety Institute for the Prevention of Occupational Accidents and Diseases (INRS), 1, Rue du Morvan, 54500 Vandoeuvre-lès-Nancy, France; 2 Institut de Recherche Robert-Sauvé en Santé et en Sécurité du Travail (IRSST), 505, Boulevard de Maisonneuve Ouest, Montreal, QC H3A 3C2, Canada; Philippe.Sarazin@irsst.qc.ca

**Keywords:** health assessment, multiple exposure, risk assessment, additivity

## Abstract

There is increasing concern for workers facing multiple chemical exposure. The accumulation of information on occupational conditions indicates the need to incorporate the concept of multiple exposures in the risk assessment process and to develop tools for assessing the potential impacts of multiple exposures on workers’ health. Our objective is to describe the MiXie online decision-making tool that can be used to assess the risk of exposure to multiple chemicals. The description includes the development of MiXie, the structure of its toxicological database according to the target organ or the mode of action, and the algorithm for quantitative analysis of a mixture. Two case studies of its use in evaluating the risks of multiple exposures in real workplace situations are presented. The case study in the printing industry showed increased risk for four toxicological classes (central nervous system damage, ocular damage, skin damage, and ototoxicity) associated with co-exposure to four chemicals during maintenance operations. The MiXie analysis also showed the presence of carcinogenic substances in the mixture and a risk to the development of the foetus. The case study in nail salons showed the presence of carcinogenic and sensitizing chemicals and an increased risk to upper airways. MiXie helps preventers evaluate the possible additive effects of mixtures, providing an easy-to-read diagnosis to identify risks incurred by co-exposed employees. In addition, MiXie identifies risky occupational situations that would go unnoticed without a multiple substance approach.

## 1. Introduction

There is increasing concern for the general population and for workers facing multiple chemical exposure. This environmental concern was first highlighted by researchers observing mixtures or cocktails of endocrine disruptors that cause a wide variety of health effects (including reproductive, metabolic, and neurological disorders) even at very low concentrations [1]. Recent findings show that mixtures of endocrine-disrupting chemicals may alter physiology and homeostasis at concentrations considered safe for the individual substances alone [2].

Multiple chemical exposures and their health effects are still the most studied combined stressor exposures. The exposure may be simultaneous or successive, as both lead to the simultaneous presence of chemicals within an organism. The most recent French national cross-sectional survey of occupational risk (SUMER), carried out in 2017, confirmed the ubiquity of multiple exposure to chemicals in France [3]. The percentage of private sector workers exposed to at least three chemicals the week before the survey has remained stable at approximately 15% since the early 2000s, with 10% of workers exposed to one or more carcinogenic, mutagenic, or reprotoxic (CMR) agents [4]. A retrospective analysis of 30,000 work situations monitored between 2005 and 2014 and recorded in two French databases also showed more than one agent present in 35% of measured situations [5]. In the USA, multiple exposure to chemicals was demonstrated in 31% of 125,551 working situations identified at 14,513 companies. Two clusters were highlighted, one for solvents (toluene, xylene, acetone, hexone, 2-butanone, and N-butyl acetate) and the other for metals (zinc, iron, lead, copper, manganese, nickel, cadmium, and chromium) [6]. In Québec, there is little data on the frequency of multiple exposures or their main characteristics. Labrèche et al. (2017) [7] estimated that 4.5% of miners and 4% of quarry workers are exposed to nickel and lead, but the data obtained by these researchers did not allow for the determination of the proportion of workers exposed simultaneously to both agents.

It remains difficult to assess the health impacts of multiple occupational chemical exposures, especially given the large number of potential combinations of substances and the different pathways involved in adverse effects [8]. Toxicological interactions resulting from pharmacokinetic or pharmacodynamic processes may occur [9]. These interactions make risk assessment difficult, since they can lead to a bigger or a smaller toxic effect than expected [10]. Given the high number of substances found in workplaces, it would be very difficult to determine the effects of all possible combinations of substances by performing experimental toxicology or epidemiological studies to obtain information about possible interactions. Several organizations, including the American Conference of Governmental Industrial Hygienists (ACGIH) and the Environmental Protection Agency (EPA), recommend that, if the toxicological effect of substances is similar (each substance affects the same target organ or has a similar effect), their combined effect can be considered the sum of their individual effects [11,12]. The additivity of effects is the most common assumption in the absence of information on the nature of the interaction, supposing that substances causing the same harmful effects would additively increase the risks to workers [13].

Historically, the “one-by-one” or “single-substance” approach has generally been used in risk prevention. However, the increase in information on occupational conditions indicates the need to incorporate the concept of multiple exposures in the risk assessment process [14]. The European Chemicals Agency (ECHA) is, accordingly, working to introduce a Mixture Assessment Factor (MAF) in REACH (Registration, Evaluation, Authorisation, and Restriction of Chemicals); this factor is to be reported in the chemical safety assessments required in the registrations of hazardous substances [15]. It is therefore necessary to develop and apply new tools for assessing the potential impacts of multiple exposures on workers’ health [16].

MiXie is an online decision-making tool that can be used to assess the risk of exposure to multiple chemicals. It works on the principle of additivity of effects (as a default assumption); that is, it supposes that substances causing the same harmful effects will additively increase the risks to workers. The sections that follow describe MiXie’s origins and functionalities as well as case studies of its use in evaluating the risks of multiple exposures in real workplace situations. The Discussion section that follows describes MiXie’s limitations and suggests an integrated prevention approach to the assessment of multiple exposure risks using MiXie.

## 2. MiXie

### 2.1. History

MiXie was developed in the late 1990s in Québec, Canada, the fruit of a partnership between Québec’s occupational health and safety research institute, the Institut de recherche Robert-Sauvé en santé et en sécurité du travail (IRSST), and the Université de Montréal. Published online for the first time in 2001, it contains toxicological information on more than 700 substances together with the occupational exposure limits (OEL) listed in Québec’s Regulations respecting occupational health and safety [17]. In 2017, toxicological experts from France’s national scientific research institute, the Institut de la recherche scientifique (INRS), adapted MiXie to France’s occupational exposure limits (8 h OEL) and improved the ergonomics of the software. The INRS, the IRSST, and the Université de Montréal have been collaborating and sharing knowledge since 2019 to develop the Québécois (https://www.irsst.qc.ca/mixie/ (accessed on 22 November 2021)) and French versions (www.inrs-mixie.fr/ (accessed on 22 November 2021)) of the MiXie tool with a common corpus of data.

### 2.2. How MiXie Works

MiXie contains a database in which toxicological effects are categorized in 24 toxicological classes according to the target organ or mode of action. For example, the effects “liver necrosis” and “contact dermatitis” are classified, respectively, as “hepatic damage” and “sensitization.” An effect can be categorized in more than one class. Table 1 shows the complete list of effects and classes.

Chemicals are listed in MiXie with their national full-shift, 8 h OEL. For each substance, the literature was reviewed to identify publications where toxic effects at relevant concentrations in the workplace were reported. Only effects at concentrations within five times the OEL in studies of humans and 100 times the OEL in animal studies were considered. Articles and general reviews were evaluated by a group of experts in toxicology, giving priority to epidemiological studies and linking toxicological classes to each substance to create the MiXie toxicological database. Additional sources of information, such as European legislation (Classification, Labelling, and Packaging regulation (CLP)) and the International Agency for Research on Cancer (IARC), were analyzed to select the toxicological effects of each substance. For example, substances belonging to IARC Group 1 (carcinogenic to humans) or Group 2A/2B (probably/possibly carcinogenic to humans) were systematically linked in MiXie to the “carcinogenic and/or mutagenic effect” toxicological class. In the same way, all substances classified H317 (may cause an allergic skin reaction) according to the CLP were linked to the “sensitization” class.

MiXie also contains an algorithm for the quantitative analysis of a mixture of chemicals. First, the toxicological classes shared by the chemicals in the cocktail are identified. Then, for each common toxicological class, the concentrations of each substance are compared with their OELs, and an exposure index is calculated for the mixture. This is called the Hazard Index (HI) (the HI is designated Rm (Mixture Ratio) and IAE (Additional Effects Exposure) in the Québécois and French versions of MiXie, respectively). If the HI is greater than 100%, the situation is considered risky for the target organ:

$$\mathrm{HI}=(\frac{C1}{\mathrm{OEL}1}+\frac{C2}{\mathrm{OEL}2}+\cdots +\frac{\mathrm{Cn}}{\mathrm{OELn}})\times 100$$

where HI is the Hazard Index, C is the observed atmospheric concentration of the substance, and OEL is its occupational exposure limit. In the French version of MiXie, the 8 h OEL of a substance is named “VLEP-8h”, and in the Québécois version, the 8 h OEL is named “VEMP”. Other values, corresponding to short-term (15 min) or ceiling OELs, may be listed when present in the regulation, but the 8 h OEL should always be chosen for the calculation of the Hazard Index when available.

MiXie can also be used for qualitative analysis when atmospheric concentrations are not available for the mixture. Both quantitative and qualitative analyses can be used to assess a work situation (i.e., identify alert classes) or a mixture (i.e., toxicological classes activated by each substance and shared classes).

The principle of additivity is applied for most toxicological classes in MiXie, with notable exceptions being the “carcinogenic and/or mutagenic effects” and “sensitization” classes (see Table 1 for complete list). For these classes, additivity is not applicable due to the nature of the effects caused and the mechanisms of action involved (referred to as “alert classes” in MiXie). Québec’s regulation respecting occupational health and safety, for example, reads as follows: When a worker is exposed to a substance with a demonstrated or suspected carcinogenic effect in humans, such exposure must be minimized, even when it falls within the standards set out in the law. From this perspective, the application of the principle of additivity for a mixture of carcinogenic substances seems a priori, contrary to the principle of avoiding any exposure that should prevail for this type of substance.

## 3. Results/Examples of Application Using the French Version of MiXie

### 3.1. MiXie in Quantitative Mode: Case Study of a Printing Workstation

**Figure 1:**
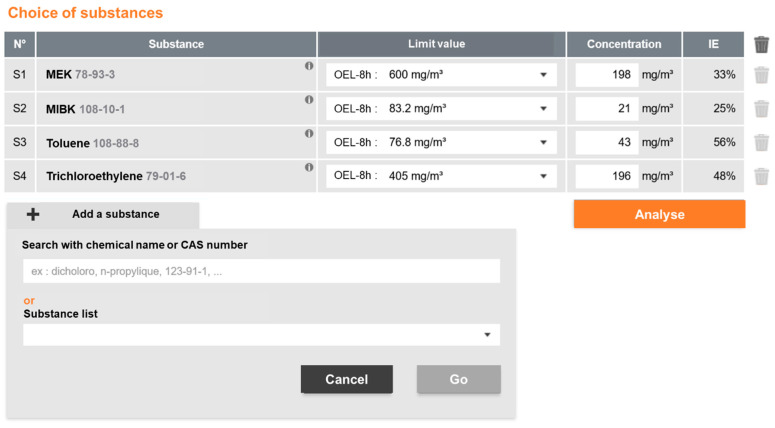
MiXie dashboard.

As part of a study of risks associated with the printing industry, industrial hygienists measured MEK (methyl ethyl ketone), MIBK (methyl isobutyl ketone), toluene, and trichloroethylene exposure during maintenance operations. Figure 1 shows the MiXie dashboard where the substances are selected and the concentration results for each substance are entered.

**Figure 2:**
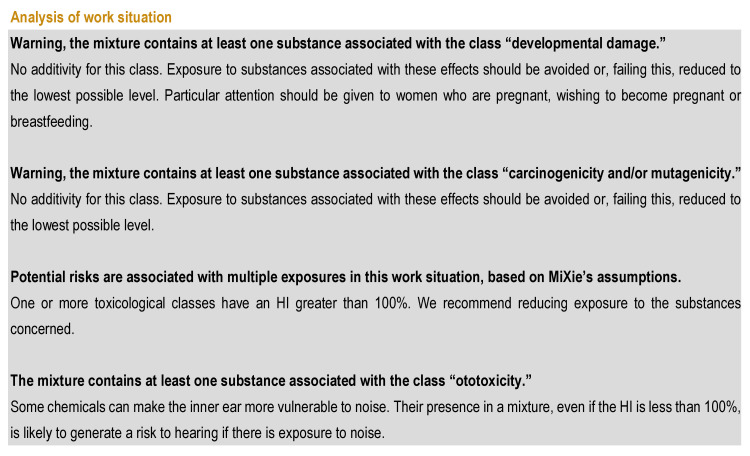
Chemical analysis of the work situation with messages linked to alert classes.

Once the substances are selected and the concentrations are entered in MiXie, a workplace chemical analysis is performed, and messages linked to alert classes and risks associated with multiple exposures are displayed (Figure 2). The messages, in this case, indicate to the user that some substances are carcinogenic, mutagenic, or reprotoxic (CMR) and/or ototoxic and that there is a risk related to multiple exposures (HI > 100%).

**Figure 3:**
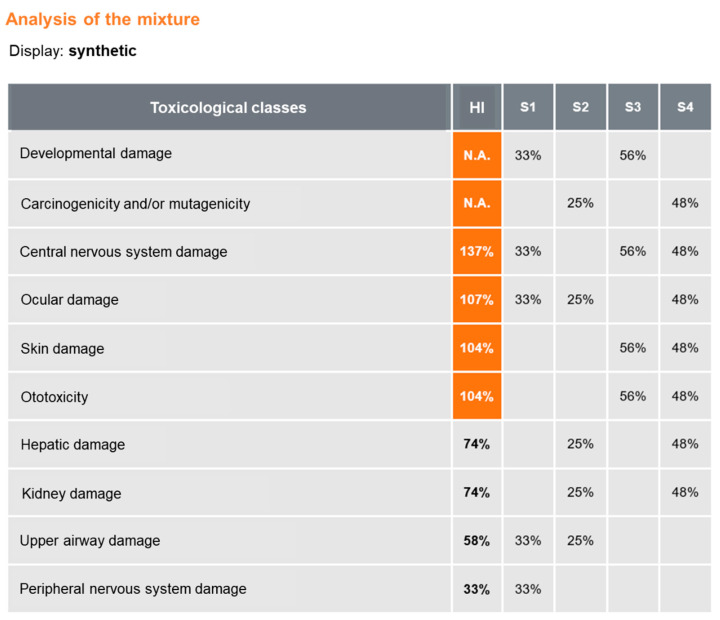
Analysis of the mixture with Hazard Index calculation (quantitative mode).

The MiXie tool then shows the details of the mixture and allows for an in-depth analysis of potential effects (Figure 3). Alert toxicological classes (for which additivity is not calculated) appear first, without calculation of the HI (N.A. = not applicable). In this example, there are two alert classes: “developmental effects on the foetus” and “carcinogenic and/or mutagenic effects.” MEK and toluene activate the former, while MIBK and trichloroethylene activate the latter. Toxicological classes listed in decreasing order of HI are then shown, with classes with an HI greater than 100% highlighted in colour.

### 3.2. MiXie in Qualitative Mode: Case Study of Nail Salons

**Figure 4:**
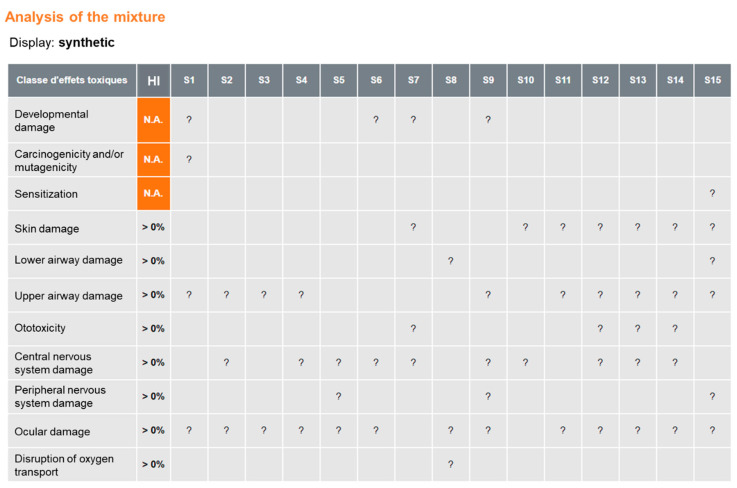
Mixture analysis without atmospheric concentrations (qualitative mode: Hazard Indexes of each toxicological class and ratios of individual substances within classes are not calculated: >0% and ?, respectively).

To illustrate the qualitative mode of MiXie, the 15 substances (S1 to S15) most frequently encountered in nail salons were selected in MiXie [18]. For qualitative analyses, MiXie shows a workplace analysis and a mixture analysis. The toxicological classes of greatest concern (i.e., those activating toxicological alert classes), along with the corresponding substances, are displayed at the top of the analysis table (Figure 4). In this case, the mixture analysis shows the following:

- Four substances affect development of the foetus, embryo, and/or child.
- One substance is carcinogenic and/or mutagenic.
- One substance is a sensitizing chemical.

MiXie then highlights classes shared by two or more substances, on the principle that the risk of affecting an organ should be considered greater when more substances activate the same class. Accordingly, the toxicological classes of concern are ocular damage (activated by 13 of the 15 substances) and upper airway damage (activated by 10 of the 15 substances). Conversely, the disruption of oxygen transport and lower airway damage concern only 1 and 2 substances of the 15, respectively.

## 4. Discussion

MiXie helps the practitioner (i.e., preventer, industrial hygienist, or occupational health physician) identify the potential additive effects of mixtures of chemicals and provides a simple signal to assess the risks of multiple exposures. It offers an easy-to-read diagnosis identifying risks incurred by co-exposed workers and gives a first level of alert, whether or not atmospheric measurements are available.

The algorithms in MiXie allow for the identification of toxicological classes associated with multiple substances based on additivity of the selected effects of each substance. The printing industry case study demonstrated that, even though no individual OELs were exceeded, the MiXie algorithms revealed four toxicological classes where the HI was >100%, a situation that poses potential health risks to workers. A single-substance approach based only on the OEL of each substance would not have identified any risk in this situation. Clerc et al. (2017) [5] analyzed multiple exposures in the French occupational exposure databank COLCHIC, focusing on the calculation of Hazard Indexes for specific health effect categories. This approach revealed HIs greater than 100% for one or more toxicological classes in close to 20% of the work situations examined, even though OELs were not exceeded. With this approach, several tasks related to health, waste treatment, or mixing were found to present higher risk than anticipated.

Both quantitative and qualitative approaches using MiXie highlight substances of greater concern (substances with CMR properties, endocrine disruptors, and sensitizers) that must be removed from the work environment and replaced if need be. The use of collective or personal protective equipment is required as well to reduce occupational exposure according to the ALARA (As Low As Reasonably Achievable) principle. In addition, the occupational physician must, depending on the activated toxicological classes, implement an appropriate, personal medical follow-up for the organs or systems identified by MiXie. Workers also need to be trained and informed about the risks of multiple exposures, especially when other deleterious factors are also present, such as biological agents, physical nuisances, psychological risks, or night work [19,20].

Though very useful, MiXie results should be interpreted with caution. The first limitation stems from use of 8 h OELs in the analysis. The OEL of a chemical substance (established by regulatory agencies) is based on the no-observed-adverse-effect level (NOAEL) or the lowest-observed-adverse-effect level (LOAEL) reported in the literature for the most critical toxicological effect in properly conducted animal and human studies. However, in calculating Hazard Indexes for a mixture, MiXie uses the same OEL for a substance for all toxicological classes, not just for the class corresponding to the toxic effect used to establish the OEL. This can lead to an overestimation of the risk for certain toxicological classes when analyzing a mixture. This is not, however, a major limitation, considering that the aim of MiXie is to provide a first level of alert.

The second limitation stems from the default hypothesis applied in MiXie, the additivity of effects. It is plausible that additivity reflects the true toxicological effect of a mixture in most cases, but it could also underestimate (supra-additivity) or overestimate (infra-additivity) the risk. Supra/infra-additivity could better represent the nature of the interaction in some cases. Infra-additivity, i.e., a less-than-additive interaction, refers to a situation in which a mixture is less toxic than the individual substances that comprise it. This beneficial interaction is sought, for example, in using ethanol to treat methanol poisoning [21]. Supra-additivity, i.e., a greater-than-additive interaction, corresponds to synergistic effects and potentialization, meaning that the mixture is more toxic than the sum of its individual components. Experimental studies show, for example, that combined exposure to noise and solvents (such as toluene) induces synergistic adverse effects on hearing [22,23]. The literature, however, includes very few reports on the nature of interactions between chemicals. A literature review conducted by Vyskocyl et al. (2014) [24] showed that the type of interaction could be established for only 11 of 218 pairs of chemicals studied. For the other pairs, the information was too fragmentary to allow for firm conclusions as to the nature of the interaction or was not confirmed by independent studies.

Finally, an overestimation of the risk may stem from the concentration selected for use in MiXie. It is recommended that personal sampling values be preferred to ambient air sampling values and that the highest value always be used when several values are available.

It is now well established that improving prevention requires consideration of multiple exposures [25]. We propose an integrated approach to risk analysis of exposure to multiple chemicals using a variety of tools. We suggest using MiXie along with tools for conducting inventories of products in workplaces (e.g., Seirich (https://www.seirich.fr/seirich-web/index.xhtml) (accessed on 22 November 2021)) and for determining chemical sampling strategies and supporting the interpretation of occupational exposure measurements (e.g., IHSTAT ([https://www.aiha.org/get-involved/VolunteerGroups/Pages/Exposure-Assessment-Strategies-Committee.aspx (accessed on 22 November 2021)), Altrex (http://www.inrs.fr/media.html?refINRS=outil13 (accessed on 22 November 2021)), and Expostats [26] (https://expostats.ca/site/info.html (accessed on 22 November 2021))).

**Figure 5:**
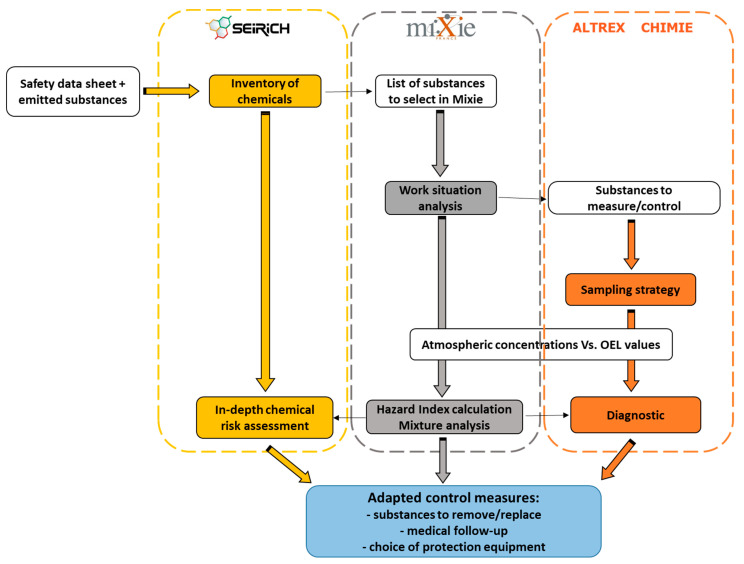
Coordinated use of MiXie, the Seirich chemical inventory, and the Altrex sampling strategy tools developed at INRS for risk assessment of multiple exposures. White boxes show input data and coloured boxes show the output generated by each tool.

Figure 5 shows a diagram of the coordinated use of various tools for multiple exposure risk assessments. Once the inventory of products used and substances emitted is completed, analysis using MiXie would identify mixtures of substances that might contribute, through the additive potential of effects selected, to hazardous effects. The results obtained would help in targeting substances to measure in a sampling campaign. MiXie would be used to analyze the mixture once atmospheric concentrations were obtained, allowing for in-depth risk evaluation and diagnostic. Practitioners would ultimately have a better understanding of the work situation and be in a better position to determine appropriate prevention and protection measures.

## 5. Conclusions

Preventing adverse health effects of occupational chemical exposure is crucial. While effects generated substance by substance are easily identifiable, those of multiple exposures are much more complicated to determine. MiXie helps preventers evaluate the possible additive effects of mixtures, providing an easy-to-read diagnosis to identify risks incurred by co-exposed employees. In addition, MiXie identifies risky occupational situations that would go unnoticed without a multiple substance approach. Taking multiple exposure into account from the very first stages of risk assessment will reduce the potentially harmful effects on workers’ health. An appropriate chemical risk assessment with coordinated use of different tools will promote risk management actions that minimize the incidence and effects of diseases related to multiple chemical exposure.

## Figures and Tables

**Table 1:** MiXie toxicological classes and associated toxic effects (# non-additive toxicological classes).

Toxicological Class	Toxic Effects	Toxicological Class	Toxic Effects
**Ocular damage**	Cataract Eye irritation Corneal edema Corneal necrosis	**Cardiovascular damage**	Cardiac damage Vascular system impairment Vasoconstriction Vasodilatation Other cardiovascular damage
**Upper airway damage**	Upper airway irritation Perforation of the nasal septum Other upper airway damage	**Autonomic nervous system damage**	Cholinesterase inhibition Muscular stimulation Other autonomic nervous system damage
**Lower airway damage**	Berylliosis Bronchitis Bronchopneumonia Pulmonary emphysema Pulmonary fibrosis Brazier’s disease Lower airway irritation Pulmonary edema Pneumoconiosis Other lower airway damage	**Disruption of oxygen transport**	Anemia Asphyxia Carboxyhemoglobinemia Blood-forming system disorder Hemolysis Cytochrome oxidase inhibition Heme synthesis inhibition Methemoglobinemia
**Central nervous system damage**	Central nervous system convulsion Central nervous system depression Other central nervous system damage	**Peripheral nervous system damage**	Peripheral neuropathy Other peripheral nervous system damage
**Hematopoietic system disruption**	Agranulocytosis Anemia Medullar aplasia Leukopenia Neutropenia Pancytopenia Thrombocytosis Thrombopenia Blood coagulation disorder	**Ototoxicity**	Cochlear damage Auditory nerve damage Vestibular damage Hyperacusis
**Metabolic acidosis**	Metabolic acidosis	**Stimulation of basal metabolism**	
**Dental or bone damage**	Bone damage Skeletal fluorosis Dental erosion Other dental or bone damage	**Skin damage**	Alopecia Chloracne Skin irritation Other skin damage (except sensitization)
**Endocrine disrupter #**	Antithyroid effect Other endocrine disrupter effect	**Male reproductive system damage #**	Testicular damage Impairment of male fertility Other male reproductive system damage
**Immune system damage**	Immune system damage	**Female reproductive system damage #**	Ovarian damage Impairment of female fertility Other female reproductive system damage
**Hepatic damage**	Liver necrosis Other hepatic damage	**Spleen damage**	Spleen damage
**Developmental damage #**	Embryonic damage Fetal damage Teratogenic effect Effect on offspring Mutagenic effect on germ cells Effect on offspring behavior Other developmental damage	**Carcinogenicity and/or mutagenicity #**	Bladder cancer Blood vessel cancer Laryngeal cancer Leukemia Liver cancer Lung cancer Mesothelioma Nasal cancer Nasopharyngeal cancer Prostate cancer Renal cancer Stomach cancer Sinonasal cancer Skin cancer Testicular cancer Upper respiratory tract cancer Mutagenic effect
**Kidney damage**	Glomerular damage Tubular damage Bladder damage Other kidney damage	**Sensitization (skin or respiratory) #**	Asthma Respiratory sensitization Contact dermatitis Skin sensitization

## Data Availability

No new data were created or analyzed in this study. Data sharing is not applicable to this article.
